# A role for Rab27 in neutrophil chemotaxis and lung recruitment

**DOI:** 10.1186/s12860-014-0039-z

**Published:** 2014-10-31

**Authors:** Rajesh K Singh, Rebecca C Furze, Mark A Birrell, Sara M Rankin, Alistair N Hume, Miguel C Seabra

**Affiliations:** Molecular Medicine, National Heart and Lung Institute, Imperial College London, London, UK; Leukocyte Biology, National Heart and Lung Institute, Imperial College London, London, UK; Respiratory Pharmacology, National Heart and Lung Institute, Imperial College London, London, UK; School of Biomedical Sciences, University of Nottingham, Nottingham, UK; Weill Cornell Medical College, 1300 York Avenue, New York, NY 10065 USA

**Keywords:** Rab27, Neutrophil, Chemotaxis, Exocytosis

## Abstract

**Background:**

Neutrophils are a critical part of the innate immune system. Their ability to migrate into infected or injured tissues precedes their role in microbial killing and clearance. We have previously shown that Rab27a can promote neutrophil migration by facilitating uropod release through protease secretion from primary granule exocytosis at the cell rear. Rab27b has been implicated in primary granule exocytosis but its role in neutrophil migration has not been investigated.

**Results:**

Here we found Rab27b to be expressed in bone marrow derived neutrophils and Rab27b knockout (Rab27b KO) along with Rab27a/b double knockout (Rab27DKO) neutrophils exhibited impaired transwell migration *in vitro* in response to chemokines MIP-2 and LTB_4_. Interestingly, no additional defect in migration was observed in Rab27DKO neutrophils compared with Rab27b KO neutrophils. *In vivo*, Rab27DKO mice displayed severe impairment in neutrophil recruitment to the lungs in a MIP-2 dependent model but not in an LPS dependent model.

**Conclusions:**

These data taken together implicate Rab27b in the regulation of neutrophil chemotaxis, likely through the regulation of primary granule exocytosis.

## Background

Neutrophils make up part of the innate immune system and provide the first line of defense against pathogens [1]. They can kill microbes in several different ways, often directly ingesting microbes or releasing microbicidal products or reactive oxygen species in their vicinity [2]. To exact these effector functions, neutrophils must first have the ability to migrate towards microbes. Several different microbial products act as chemoattractants for neutrophils, such as formyl-met-leu-phe (fMLF). In addition, other leukocytes such as macrophages/monocytes and mast cells activated by inflammatory signals or microbial products can secrete chemoattractants such as macrophage inflammatory protein 2 (MIP-2) and leukotriene B_4_ (LTB_4_) that can cause neutrophils to extravasate into injured or infected tissues. Therefore the ability of neutrophils to migrate effectively is critical to bring them into proximity to microbes to allow anti-microbial activity. Neutrophils are short-lived cells and are released from the bone marrow into the blood stream. Upon infection, neutrophils are released from the bone marrow into the blood stream in large numbers through the actions of chemokines KC and MIP-2 (that act on receptor CXCR2) and granulocyte colony stimulating factor (G-CSF) that reduces bone marrow retention of neutrophils by down-regulating chemokine (C-X-C motif) receptor 4 (CXCR4) expression [3]. Only KC and MIP-2 mediate recruitment of neutrophils into tissues.

At the site of infection or injury, tissue resident cells produce chemokines such as MIP-2 and this creates a chemokine gradient with increasing concentrations leading to the site of infection [3]. Neutrophils sense very low concentrations of chemokine via binding of chemokine to chemokine receptors on their surface such as MIP-2 binding to receptor CXCR2 [4]. This triggers a signaling cascade that stimulates actin polymerization and polarization of the cell. The neutrophil continues to migrate under this chemokine gradient. The process of actin polymerization is essential to the migration process because it promotes formation of lamellipodia and filopodia by pushing the membrane forward at the leading edge of the cell. This is essential for movement and is driven by Rho GTPases Cdc42, Rac1, Rac2 and the Rac and Cdc42 GEFs p-Rex1 and Vav1 that activate Rac and Cdc42 [5-7]. At the trailing edge, the cell detaches and retracts its uropod through a calcium dependent process involving the activities of RhoA, RhoA kinase ROCK and myosin heavy chain IIA [8-12]. Recent studies, including from our own group, have suggested the action of exocytic mediators such as Synaptotagmin VII and Rab27a to promote uropod release by delivering proteases important for de-adhesion to the cell rear and therefore allow efficient migration [13,14].

Rab27 is a Rab family GTPase that has been implicated to promote regulated exocytosis of lysosome related organelles (LROs) in a wide variety of secretory cells including CD4^+^ and CD8^+^ lymphocytes [15,16], neutrophils [17], natural killer (NK) cells [16,18], mast cells [19,20] and platelets [21]. Rab27 exists in two isoforms, Rab27a and Rab27b that share roughly 70% protein sequence similarity. While previously thought to play the same role, recent studies have indicated that in cells that co-express both isoforms, such as HeLa cells, neutrophils, platelets and mast cells, that they may play different roles, though the basis of this is yet to be established [20-23]. In neutrophils, Rab27a and Rab27b were both found to promote primary granule exocytosis though it was proposed by independent mechanisms [23].

When active, Rab27 is present on the membrane of LROs and can bind to a distinct family of effector proteins to mediate functions such as binding to cortical actin. This can lead to peripheral retention of LROs [24] or LRO docking at the plasma membrane prior to a fusion event [25]. We had previously hypothesized that primary granule secretion at the rear of the cell was important for uropod release and efficient migration. Rab27b had been previously shown to regulate primary granule secretion from neutrophils but its role in neutrophil migration had not been studied. Therefore the purpose of this study was to investigate whether in addition to regulating primary granule release, it might also regulate neutrophil migration.

## Results

### Rab27b is expressed in BMDN and Rab27b KO and Rab27DKO neutrophils display impaired transwell migration *in vitro*

**Figure 1:**
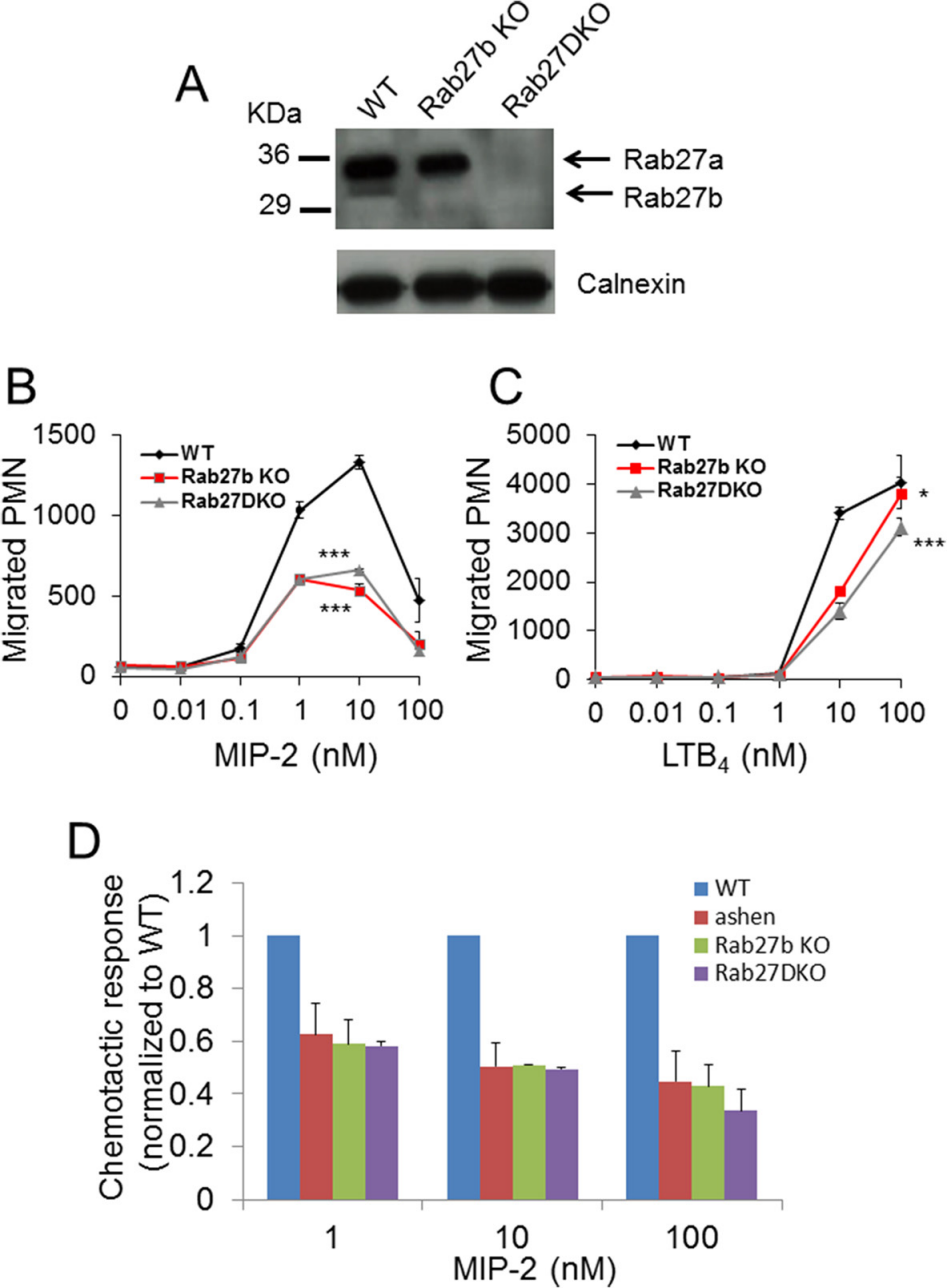
**Rab27a and Rab27b promote neutrophil chemotaxis** ***in vitro*** **. (A)** Immunoblot analysis of Rab27a and Rab27b expression using 50 μg lysates from bone marrow derived neutrophils (BMDN) purified from wild-type, *Rab27b* KO and Rab27DKO mice. Antibody recognized both Rab27a (upper band) and Rab27b (lower band). Transwell migration of BMDN from wild-type, Rab27b KO and Rab27DKO across a range of concentrations of **(B)** MIP-2 and **(C)** LTB4 after 30 min. **(D)** Comparison of transwell migration of wild-type, ashen, Rab27b KO and Rab27DKO BMDN using MIP-2 after 30 min. * *p* ≤0.05, *** *p* ≤0.001 using ANOVA. Error bars s.e.m. Data representative of 3 similar experiments.

We previously showed that Rab27a regulates neutrophil responses to chemoattractants MIP-2 and LTB_4_ and also to bacterially derived peptide fMLF [14], however the role of Rab27b in this process was not analyzed. To examine the role of Rab27b in neutrophil migration, we first confirmed expression of Rab27b in BMDN. To do this, we purified neutrophils from bone marrow of wild-type, Rab27b KO and Rab27DKO mice and subjected lysates to SDS-PAGE and immunoblotting using an antibody that recognized both Rab27a and Rab27b isoforms. Wild-type BMDN displayed expression of both Rab27a (upper band) and Rab27b (lower band) (Figure 1A). The slight difference in apparent size of Rab27a and Rab27b has been observed previously [20,26]. Rab27b KO BMDN expressed only Rab27a (upper band) and Rab27DKO BMDN expressed neither Rab27a nor Rab27b. Next we used a transwell migration assay to examine the *in vitro* chemotaxis of Rab27b KO BMDN. Both *Rab27b* KO and Rab27DKO neutrophils displayed significantly impaired transwell chemotaxis compared with wild-type neutrophils using MIP-2 (Figure 1B) and LTB_4_ (Figure 1C) to stimulate chemotaxis. Interestingly, a comparison of Rab27a deficient (*ashen*), Rab27b KO and Rab27DKO neutrophil chemotaxis in response to MIP-2 suggested that there is a similar level of impairment in all 3 mutant neutrophil types using three different concentrations of MIP-2 (Figure 1D). This indicates a requirement for both Rab27a and Rab27b in neutrophil chemotaxis and that the two isoforms may be mutually dependent on each other to promote chemotaxis. Intriguingly these results mirror those observed for primary granule exocytosis from Rab27a, Rab27b and Rab27DKO neutrophils [23]. In that case, Rab27a and Rab27b were proposed to play independent roles in exocytosis.

### Deficiency of Rab27 does not perturb surface expression of chemokine receptor CXCR2

**Figure 2:**
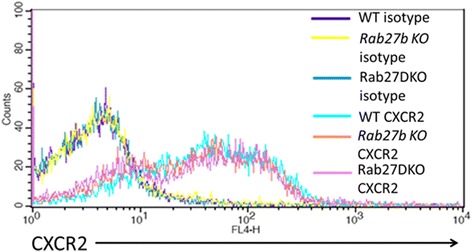
**Surface CXCR2 expression is comparable on BMDN from wild-type, Rab27b KO and Rab27DKO mice.** Flow cytometry analysis of surface CXCR2 expression on purified wild-type, Rab27b KO and Rab27DKO BMDN. Isotype control was used to assess non-specific antibody binding.

Depletion of Rab27b has previously been suggested to reduce surface expression of c-kit in megakaryocytes [27]. To rule out the possibility that deficiency of Rab27b might impair chemokine receptor expression, we analyzed CXCR2 (receptor for MIP-2) expression on wild-type, Rab27b KO and Rab27DKO BMDN by flow cytometry. Similar levels of surface CXCR2 were observed on all neutrophils (Figure 2) suggesting that reduced receptor expression does not explain impaired chemotaxis of Rab27b KO and Rab27DKO neutrophils.

### Rab27 regulates neutrophil recruitment in a MIP-2 driven model of lung inflammation *in vivo*

**Figure 3:**
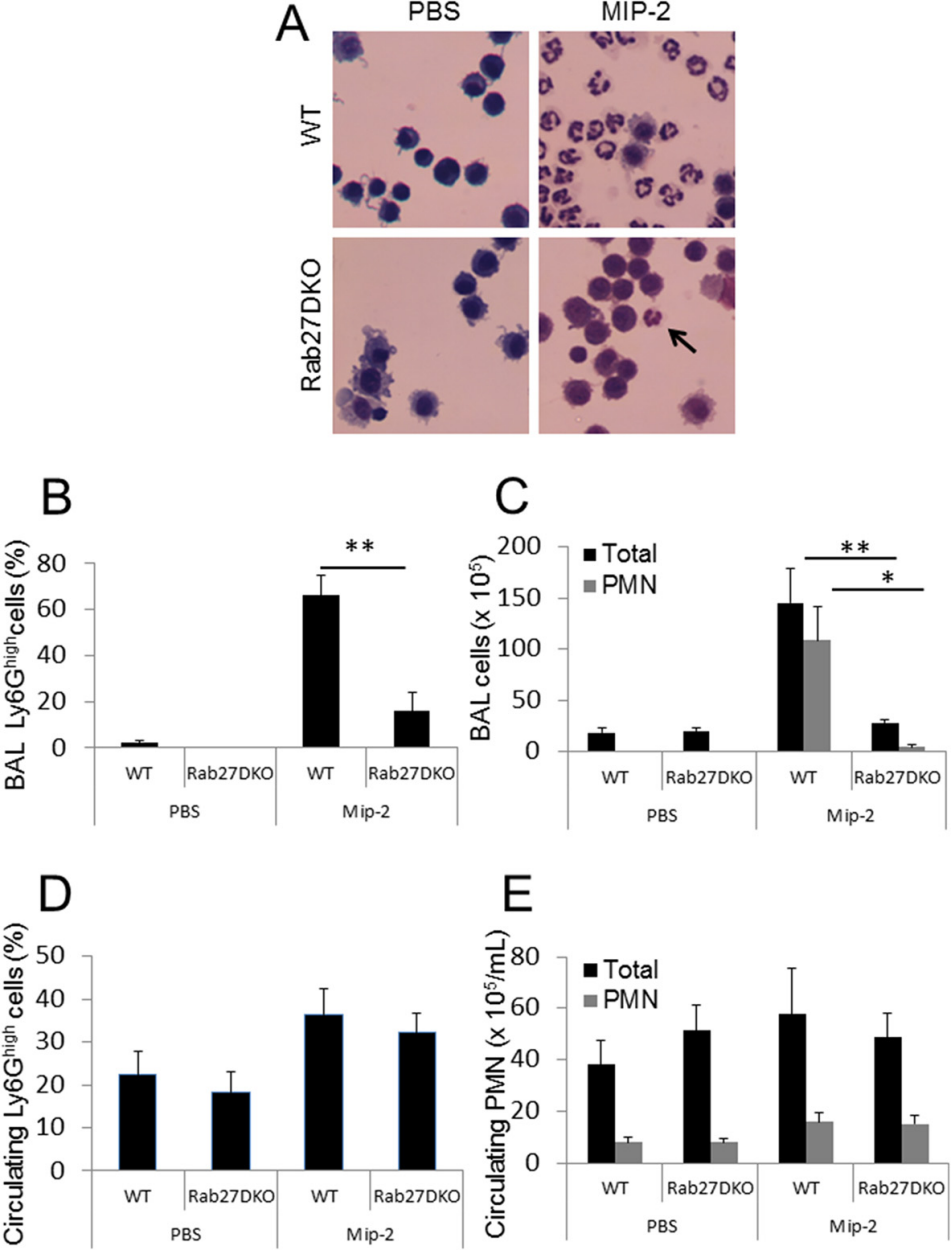
**Rab27 promotes MIP-2 dependent neutrophil recruitment to the lungs** ***in vivo*** **.** Wild-type (WT) and Rab27a/b double knockout (Rab27DKO) mice were treated with PBS vehicle or MIP-2 via intranasal administration and 2 hours after treatment, bronchoalveolar lavage (BAL) **(A-C)** and blood cells **(D, E)** were collected. BAL cells from wild-type and Rab27DKO mice treated with PBS and MIP-2 were collected, cytospun and differentially stained **(A)**. Shown are representative images of BAL cells showing presence of neutrophils in MIP-2 treated WT mice and greatly reduced numbers in Rab27DKO mice (arrow). BAL cells were stained for Ly6G to quantify percentage neutrophil content by flow cytometry **(B)**. Total cells and neutrophils collected from BAL of wild-type and Rab27DKO mice treated with PBS and MIP-2 were quantified **(C)**. Circulating cells in blood drawn from PBS or MIP-2 treated wild-type and Rab27DKO mice were stained for Ly6G to assess proportion of total cells that were neutrophils by flow cytometry **(D)** and total numbers of circulating neutrophils could be quantified **(E)**. * *p* ≤0.05, ** *p* ≤0.01 student’s t test. Error bars s.e.m. Data from PBS treated mice (n = 5 for wild-type and Rab27DKO) and MIP-2 treated mice (n = 5 for wild-type and n = 6 for Rab27DKO).

We have previously shown that Rab27a promotes MIP-2 dependent neutrophil recruitment *in vivo* [14] and proposed that this occurs by stimulating primary granule exocytosis at the cell rear to facilitate uropod release to aid cell migration. Rab27b has recently been implicated in primary granule exocytosis [23]. To understand whether Rab27b may contribute to the process of neutrophil recruitment *in vivo*, we challenged Rab27a/b double knockout (Rab27DKO) mice with MIP-2 intranasally. This model was chosen for its ability to stimulate neutrophil recruitment independently of other cell types. This is beneficial because other cell types also express Rab27a and/or Rab27b and deficiency of Rab27 has been associated with changes in regulated secretion from many cell types that may influence the recruitment of neutrophils in vivo. In vehicle PBS treated mice, neutrophil and total cell numbers in the BAL were low and similar numbers were observed between wild-type and Rab27DKO mice (Figure 3A,B). When mice were challenged with MIP-2, robust neutrophil recruitment was observed in wild-type mice but a significantly reduced response was observed in Rab27DKO mice. The percentage of BAL cells that were neutrophils was 66.04 ± 8.67% in wild-type mice vs 15.61 ± 8.34% in Rab27DKO mice (Figure 3A,B). Quantification of total and neutrophil numbers in the BAL showed that in wild-type mice, the total number of cells in the BAL increased significantly upon MIP-2 treatment and that most of these cells were neutrophils (wild-type PBS treated 0.19 ± 0.14 × 10^6^ vs 11.82 ± 3.19 × 10^6^ wild-type MIP-2 treated) (Figure 3C). However the total number of BAL cells and neutrophil numbers did not significantly increase in Rab27DKO mice upon MIP-2 treatment (Rab27DKO PBS treated 0.02 ± 0.01 × 10^6^ vs 2.25 ± 1.59 × 10^6^ Rab27DKO MIP-2 treated) (Figure 3C). Comparable increases in neutrophil percentages and total neutrophil numbers in the blood were observed in wild-type and Rab27DKO mice in response to MIP-2 treatment (Figure 3D,E). This indicates that mobilization of neutrophils into the circulation from bone marrow in response to MIP-2 was unperturbed in Rab27DKO mice and that the defect in neutrophil recruitment in Rab27DKO mice is from the blood to the alveolar space.

### LPS allows by-pass of Rab27 function in neutrophil recruitment to the lungs *in vivo*

**Figure 4:**
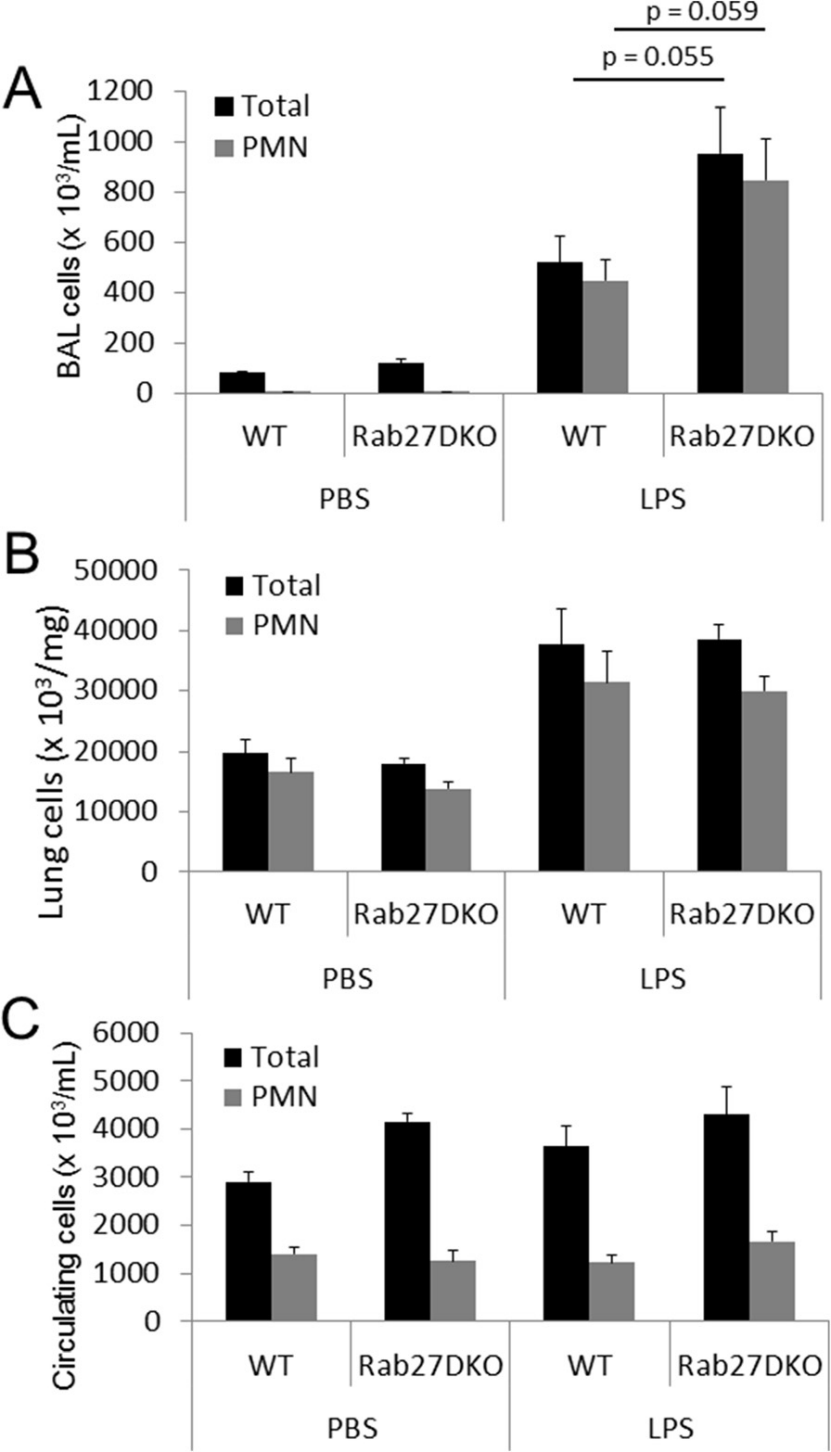
**Rab27 does not regulate LPS dependent neutrophil recruitment to the lungs** ***in vivo*** **.** Wild-type (WT) and Rab27a/b double knockout (Rab27DKO) mice were treated with PBS vehicle or LPS and 6 hours after treatment bronchoalveolar lavage (BAL) **(A)**, lung tissue **(B)** and blood cells **(C)** were collected and total and neutrophil numbers quantified by differential staining. Error bars s.e.m. P values student’s t test. Data from PBS treated mice (n = 8 for wild-type and Rab27DKO) and LPS treated mice (n-8 for wild-type and n = 9 for Rab27DKO).

Under physiological conditions, neutrophils are recruited to the lungs in response to injury or infection. This stimulates the release of an array of pro-inflammatory cytokines by lung resident macrophages and other cells, which promote the recruitment of neutrophils to the lungs. This stimulus is much more potent than simply using MIP-2 as in Figure 3. Therefore to better represent a physiological model of lung inflammation and to stimulate robust recruitment of neutrophils to the lungs, we utilized a model of LPS induced lung inflammation. We challenged wild-type and Rab27DKO mice with aerosolized PBS or LPS and 6 h post-challenge mice were assessed for neutrophil recruitment to the BAL, lung and numbers in circulation analyzed. LPS treatment stimulated increases in total cell and neutrophil numbers in the BAL in both wild-type and Rab27DKO mice compared with PBS treated control mice (Figure 4A,B). While an increase in total cell and neutrophil numbers in the BAL was observed in LPS treated Rab27DKO mice compared with LPS treated wild-type mice, this was not statistically significant. Analysis of total cell and neutrophil numbers in circulation also did not reveal differences in wild-type and Rab27DKO mice (Figure 4C). Taken together these data indicate that in an LPS driven model, Rab27 does not regulate neutrophil recruitment to the lungs. LPS treatment represents a more biologically complex model of inflammation and it may be that changes in secretion of pro-inflammatory cytokines by other cell types may compensate for impaired chemotaxis in Rab27 knockout neutrophils. A recent study analyzed the role of Rab27a in lung infiltration of neutrophils [28]. Using a systemic LPS driven model of inflammation they found no defect in neutrophil recruitment to the lungs in *ashen* mice. This is in contrast to our previous findings that Rab27a can regulate neutrophil recruitment to the bronchoalveolar lavage using a MIP-2 dependent model [14]. The disparity in these results suggests the possibility that LPS might bypass Rab27 dependent regulation of neutrophil recruitment *in vivo*.

## Discussion and conclusion

In this study, we found that Rab27DKO mice displayed severe impairment in neutrophil recruitment to the BAL in a MIP-2 dependent model of neutrophil recruitment but not in an LPS dependent model of neutrophil recruitment. This raises the possibility that LPS may somehow bypass Rab27 dependent regulation of neutrophil chemotaxis in vivo. Others have shown that using an LPS induced model of acute lung injury, inhibition of CXCR2 by Reparixin reduces neutrophil recruitment to the lungs by 50% [29] and a similar level of impairment was observed in CXCR2 knockout mice [30]. Using a dual inhibitor of CXCR1/2 Sch527123, LPS induced neutrophil recruitment was observed to be reduced by 75%, suggesting that CXCR1 can also contribute to neutrophil recruitment in an LPS stimulated model [31]. Therefore more than one receptor is responsible for LPS induced neutrophil recruitment. A role for Rab27 exclusively in CXCR2 mediated neutrophil recruitment is unlikely given that in this study Rab27b KO and Rab27DKO BMDN displayed impaired chemotaxis in response to LTB_4_ as well as MIP-2. A likely explanation for the difference may be in the production of pro-inflammatory cytokines in response to LPS by macrophages and the endothelium that causes neutrophil recruitment in LPS dependent models. Rab27a deficient *ashen* mice develop haemophagocytic lymphohistiocytosis (HLH) caused by uncontrolled macrophage activation upon LCMV infection and Rab27 deficient macrophages are predisposed to hyper-activation. Therefore upon LPS stimulation it is possible that they may secrete higher levels of proinflammatory cytokines such as KC and MIP-2 and this may be enough to compensate for a deficiency in uropod release. As mentioned previously, other cell types also express Rab27a/b that may influence neutrophil recruitment. Deficiency of Rab27a increases von-Willebrand Factor secretion from endothelial cells [32], which promotes leukocyte rolling, adhesion and recruitment to tissues. This potentially could promote neutrophil recruitment to the lungs independently of neutrophil function.

It is also possible that LPS and MIP-2 promotes neutrophil use of integrins that are different to allow lung recruitment. We showed previously that MIP-2 stimulated surface Cd11b levels are downregulated in wild-type cells but not in Rab27a KO cells. We suggested the reason for this was impaired protease release from Rab27a KO neutrophils that delays cleavage of Cd11b and inhibits uropod release [14]. Interestingly, neutrophils elicited by *E. coli* to the lungs do not require Cd11/Cd18 for lung recruitment [33]. This suggesting that *E. coli* (and *E. coli* derived LPS) may promote neutrophil usage of integrins that is not regulated by Rab27.

**Figure 5:**
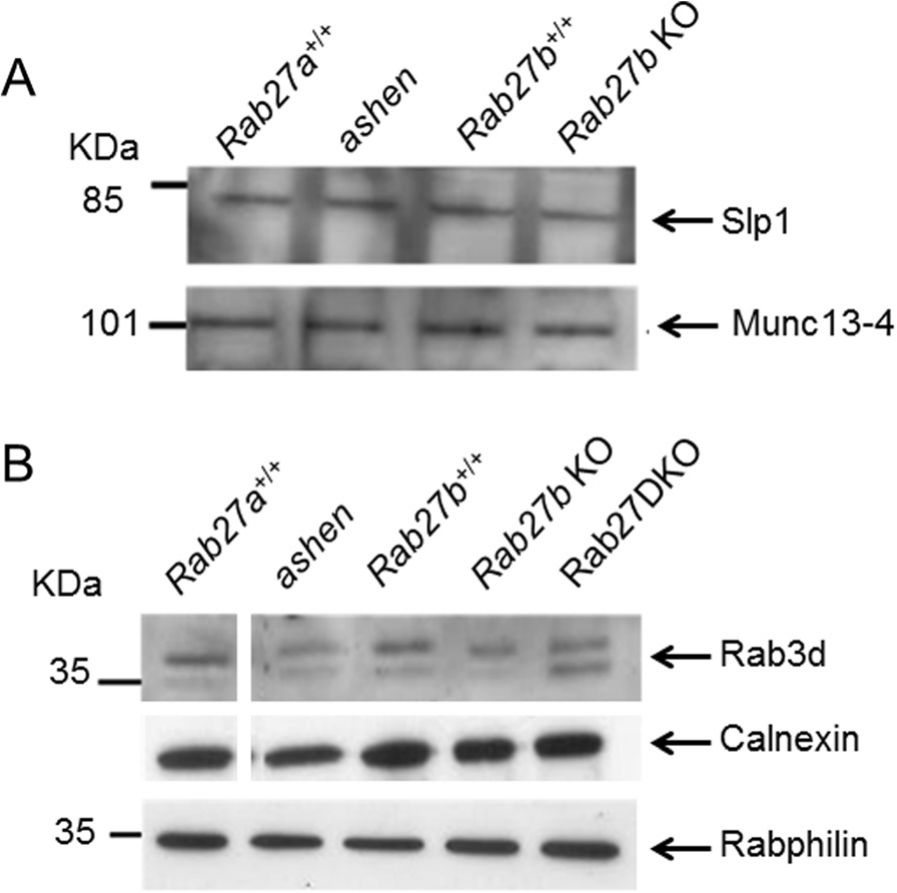
**Slp1, Munc13-4, Rab3d and Rabphilin are expressed in BMDN. (A)** Immunoblot analysis of Slp1 and Munc13-4 expression using 100 μg lysates from BMDN purified from wild-type (*Rab27a* ^+/+^, *Rab27b* ^+/+^), ashen and Rab27b KO mice. **(B)** Immunoblot analysis of Rab3d and Rabphilin expression using 100 μg lysates from BMDN purified from wild-type (*Rab27a* ^+/+^, *Rab27b* ^+/+^), ashen and Rab27b KO and Rab27DKO mice. Calnexin was used as a loading control.

Comparison of BMDN transwell chemotaxis shows that Rab27a and Rab27b deficient BMDN display the same level of impairment. This suggests that both Rab27a and Rab27b are required for normal responses. Furthermore knockout of both Rab27a and Rab27b does not additionally impair chemotaxis and Rab27DKO BMDN retain approximately 50% of wild-type chemotactic response. In *ashen* BMDN, a delay in uropod release is observed [14]. This suggests that other proteins may be present in neutrophils that are able to promote migration, possibly by allowing primary granule release at the back of the cell to aid uropod detachment in absence of Rab27. Preliminary evidence suggests that Rab27 effectors Slp1, Munc13-4, Rab27 related protein Rab3d, and shared Rab27/Rab3 effector Rabphilin are expressed in BMDN (Figure 5A and 5B). This raises the possibility in the presence of Rab27, it may act with Slp1 and Munc13-4. Rab3d may compensate in absence for Rab27a/b to restore some primary granule exocytosis and chemotaxis to neutrophils. Further investigation is required to confirm this.

How Rab27 (and potentially Rab3) work with effectors and what the interplay between Rab27a and Rab27b is in the same cell is an active area of interest. Several models have been proposed by which Rab27a and Rab27b can act within the same cell [34]. As Rab27a and Rab27b KO neutrophils display the same level of impaired migration (Figure 1D), it may indicate that Rab27a and Rab27b act in the same pathway, possibly in tandem to promote primary granule secretion and chemotaxis. This may occur through interaction with different effectors, as has been suggested previously [20] or by different localizations of Rab27a and Rab27b. This has been shown previously in neutrophils where Rab27a is located on primary and other cytoplasmic granules whereas Rab27b is more peripherally distributed [23]. This suggests a model where Rab27a acts upstream to deliver granules to the periphery of the cell and Rab27b is required for late stage primary granule fusion with the plasma membrane. Further studies are required to understand whether Rab27a and Rab27b function with the same effectors or different effectors within the same cell and how this is able to occur.

Our previous study indicated that Rab27a KO neutrophils display a uropod retraction defect due to impaired released of proteases, likely from primary granule exocytosis [14]. Further studies are required to understand whether Rab27b KO neutrophils display the same phenotype. It has been shown previously that primary granule exocytosis is required for cleavage of integrin Cd11b which promotes uropod release and efficient migration in neutrophils [35]. A previous study indicated that Rab27b KO and Rab27DKO neutrophils display a similar level of impaired primary granule exocytosis as Rab27a KO neutrophils [23]. This may indicate that both Rab27a and Rab27b promote uropod release through primary granule exocytosis and release of proteases that cleave Cd11b and promote uropod release. The lack of an additive effect of Rab27a and Rab27b knockout may also indicate independent functions of the two isoforms in exocytosis. Further studies are necessary to test these ideas.

## Methods

### Mouse strains and purification of neutrophils

Wild-type C57BL/6, Rab27a^+/+^, Rab27b^+/+^, *ashen* Rab27a^ash/ash^, Rab27b knockout (Rab27b^−/−^) and Rab27a/b double knockout (Rab27DKO) mice were bred in-house and generated as described previously [21,26]. All animal experiments were performed with the approval of an ethics committee and in compliance with the UK Home Office Regulations under PPL 70/7078 at the Central Biomedical Sciences of Imperial College, London, UK. Neutrophils from mouse bone marrow were purified as described previously [36]. In brief, bone marrow cells were flushed from femurs and tibias of mice with PBS and red blood cells lysed by resuspension in a solution of 0.168 M NH_4_Cl, 10 mM KHCO_3_ and 0.097 mM ethylenediaminetetraacetic acid (EDTA). Cells were washed once with phosphate buffered saline (PBS), resuspended in 1 mL of PBS and layered on top of a discontinuous Histopaque gradient containing 3 mL of Histopaque (Sigma-Aldrich) at 1.119 g/mL at the bottom and 3 mL of Histopaque at 1.077 g/mL on top. Cells were centrifuged for 45 min at 700 g in a swing bucket centrifuge without braking. Cells at the interface of the two layers were collected and washed twice with PBS. Typical preparations contained above 80% neutrophils as assessed by Ly6G^high^ staining by flow cytometry.

### Antibodies, immunoblot and flow cytometry

For immunoblot analysis, S087 rabbit immune serum was used to detect both Rab27a and Rab27b. Slp1 and Munc13-4 rabbit immune serum used for immunoblot were kindly provided by Dr. Sergo D. Catz (The Scripps Research Institute, La Jolla, CA, USA) and were described previously [17,37]. Immunoblots were performed as described previously [14]. For flow cytometry using a FACScalibur cytometer, phycoerythrin (PE)-conjugated anti-Ly6G (BD Biosciences) and allophycocyanin (APC)-conjugated CXCR2 (R&D Systems) antibodies were used at 1:100 dilution. For surface antigen staining, 2 × 10^5^ bone marrow derived neutrophil (BMDN) cells were washed twice and resuspended in rat anti-FcγRII/RIII antibody (BD biosciences) for 10 minutes, then incubated with fluorophore-conjugated antibodies for 30 minutes on ice.

### Transwell migration assay

Neutrophils (1 × 10^5^) from wild-type and knockout mice were resuspended in 100 μL of Roswell Park Memorial Institute (RPMI) medium containing 1% bovine serum albumin (BSA) and placed on top of a chemotaxis plate with 3 μm pore diameter (Receptor Technologies, Adderbury, UK) and allowed to migrate towards recombinant murine MIP-2 (Peprotech) or LTB_4_ (Sigma-Aldrich) at indicated concentrations at 37°C for 30 min. Cells that had migrated through the pores to the lower chamber were collected, resuspended in 200 μL volume, placed on ice and counted for 30 seconds on the high setting using a FACScalibur system (BD Biosciences).

### *In vivo* MIP-2 dependent neutrophil recruitment assay

The recruitment of neutrophils to the lungs using MIP-2 was performed as described previously [38] with the following modifications. Mice were briefly anaesthetised with isofluorane and 0.8 μg of recombinant murine MIP-2 (Peprotech) or PBS was administered intranasally. 2 hours after challenge, mice were euthanized and the bronchoalveolar lavage (BAL) and blood collected. BAL samples cytospun onto glass slides and blood smears were stained using Diff-Quik staining kit and neutrophil percentages measured by FACS. Total cells were counted manually using a haemocytometer. Neutrophils were identified as Ly6G^high^ cells and by their characteristic high-side-scatter profile.

### *In vivo* LPS dependent neutrophil recruitment assay

The recruitment of neutrophils to the lungs using LPS was as described previously [39]. In brief, mice were challenged with aerosolized PBS or 1 mg/mL LPS (*Escherichia coli*, serotype 0111:B4, Sigma-Aldrich Ltd. Poole, UK) for 30 min. 6 h post-challenge mice were euthanized and BAL collected. The lungs were collected and digested using collagenase as described previously [40]. Cells were cytospun onto glass slides and differential counts obtained by staining using Wright-Giemsa.
